# Identifying the superior antibiotic prophylaxis strategy for breast surgery: A network meta-analysis

**DOI:** 10.1097/MD.0000000000015405

**Published:** 2019-04-26

**Authors:** Tao Guo, Baiyang Chen, Fengying Rao, Ping Wu, Pengpeng Liu, Zhisu Liu, Zhen Li

**Affiliations:** a Department of Hepatobiliary and Pancreatic Surgery, and Department of General Surgery, Zhongnan Hospital of Wuhan University, Wuhan; b School of Nursing, Huanggang Polytechnic College, Huanggang, P.R. China.

**Keywords:** antibiotic prophylaxis, breast surgery, network meta-analysis

## Abstract

**Background:**

The clinical roles of different antibiotic prophylaxis strategies for breast surgery remains large unknowns. The aim of this study is to evaluate different antibiotic prophylaxis strategies based on a network meta-analysis.

**Methods:**

We initially retrieved literature from globally recognized databases, namely, MEDLINE, EMBASE and Cochrane Central, to address relative randomized controlled trials (RCTs) investigating the antibiotic prophylaxis strategies for breast surgery. Relative postoperative infection rates were quantitatively pooled and estimated based on the Bayesian theorem. Values of surface under the cumulative ranking curve (SUCRA) probabilities were calculated and ranked. Additional pairwise meta-analyses were performed to validate differences between the respective strategies at the statistical level for further explanations.

**Results:**

After a detailed review, a total of 14 RCTs containing 6 different strategies were included for the network meta-analysis. The results indicated that the application of antibiotics administered during pre- plus post- plus intraoperative periods possessed the highest possibility of having the best clinical effects (SUCRA, 0.40), followed by intraoperative administration alone (SUCRA, 0.35) and pre- plus intraoperative administrations (SUCRA, 0.20). Moreover, an additional pairwise meta-analysis determined that pre- and intraoperative-related strategies significantly reduced postoperative infections at a statistical level.

**Conclusion:**

Based on the current evidence, we concluded that application of antibiotics administered during pre- plus post- plus intraoperative periods seemed to reveal superior benefits. However, the essential roles of pure intraoperative and postoperative application were still need to be further validated.

## Abstract 2

Supplemental Digital Content is available in the text

## 1 Introduction

Surgery involves a risk of postoperative infection, which has been shown to be one of the most common adverse events in hospitalized patients.^[1]^ Any surgical procedure carries the risk of infection, which can delay subsequent adjuvant treatment, and severe infection may cause poor morbidity and high cost of treatment.^[2,3]^ On the contrary, surgeries are often regarded as clean, clean-contaminated, or contaminated operations. Clean surgery normally involves an uninfected wound, where no inflammation is encountered and the respiratory, alimentary, genital, and urinary tracts are not involved; usually, clean surgery has a low risk of contamination with a predicted surgical site infection (SSI) rate of 1.5% to 3.5%.^[4–6]^ With the development of surgical sterilization and minimally invasive procedures, the postoperative infection rate should gradually reduce. However, in reality, the postoperative infection rate of breast surgeries is approximately 30%, which is completely inconsistent with theoretical expectations due to the breast surgical procedure being regarded as a clean surgery.^[7–9]^ Therefore, antibiotic prophylaxis is wildly applied for breast surgical procedures, aiming to reduce the incidence of postoperative infections, which would mean less morbidity for patients and less antibiotic pressures on the environment.

In the last 3 decades, many randomized controlled trials investigating the efficacy of antibiotic prophylaxis on breast surgeries were published from different regions with contradictory results. Furthermore, although these trials tried to discover clinical effects of antibiotic prophylaxis, they were conducted with multifarious strategies. More importantly, until now, no consensus on the superior prophylactic antibiotic strategy for breast surgery and no quantitative network estimation were conducted to comprehensively evaluate these antibiotic prophylaxis strategies. Therefore, it is necessary to perform a quantitative network meta-analysis to identify the superior antibiotic prophylaxis strategy for breast surgery to provide complete clinical evidence. Meanwhile, this study also undertook the purpose of raising the directions of future clinical research.

## 2 Methods

### 2.1 Literature search and retrieval

Current meta-analysis was based entirely on previous published studies that had declared ethical approvals and no original clinical raw data were collected or utilized, thereby ethical approval was not conducted for this study. Our review was performed according to previously established Preferred Reporting Items for Systematic Reviews and Meta-Analyses (PRISMA) guidelines^[10]^ and was pre-registered in PROSPERO (CRD42019121077). The retrieval of data for this study was initialized in globally recognized electronic databases, including MEDLINE, EMBASE, and Cochrane Central, to avoid regional bias and to achieve optimal raw data. Relative mesh items and their combinations were applied to address relevant trials investigating antibiotic prophylaxis for breast surgeries (Example retrieval strategy of MEDLINE is presented in Supplementary Table S1). One requirement was that the text needed to be in English; however, the publication status and date were not restricted.

### 2.2 Inclusion and exclusion criteria

Two researchers independently reviewed the title and abstract of each study to select those that were likely included for further screening if they met the following criteria: randomized controlled trials (RCTs); studies comparing different antibiotic prophylaxis strategies for breast surgeries; and studies providing available parameters of interest. Meanwhile, the following items were defined as exclusion criteria: non-RCTs; no available parametric data reported; studies focusing on basic science or other surgeries; reviews, case reports or comments; and vague or same strategies.

### 2.3 Outcome selection

In the current study, we aimed to evaluate the clinical efficacy of different antibiotic prophylaxis strategies. Thus, the postoperative infection rate was selected as the parametric data. The raw data of relative dichotomous variables were regarded as outcome of interest. Data could be described as any signs and symptoms related to infections, including local (such as SSI) and systematic (such as systemic inflammatory response syndrome) infection events.

### 2.4 Raw data extraction and quality assessment

General information (e.g., author name, publication data and region) and intervention-related characteristics (e.g., sample size and reported parameters) were recorded using a predesigned form. For raw data of outcome with interest, the overall rate of total infections was extracted as the parametric data for the final pooled estimation.

On the contrary, we only included RCTs for the current study; therefore, we applied the Cochrane Risk of Bias assessment tool^[11]^ to evaluate the bias risk of individual studies with the following requirements: free of selection bias; free of performance bias; free of detection bias; free of attrition bias; free of reporting bias; and free of other biases. A graphic summary of the overall and study-level risk of bias was made using Review Manager Software (Version 5.3, Cochrane Community, United Kingdom).

The raw data extraction and bias risk assessment were independently conducted by 2 investigators. Any disagreements were resolved by a group discussion with all team members.

### 2.5 Certainty of evidence

To confirm the reliability and quality of the current study, the Grades of Recommendations Assessment, Development, and Evaluation (GRADE) criteria were selected to assess the methodological quality of evidence.^[12]^ Relative items (such as research limitations, inconsistent findings, uncertain direct evidence, possible confounding factors, publication bias, and so on) were considered for evidence rating. Moreover, in conditions of existing direct and indirect comparisons, higher grade would be considered as the final recommendations for network meta-analysis.^[13]^ All investigators assessed the grade of the examined studies through discussion until reaching agreement.

### 2.6 Statistical analysis

We aimed to comprehensively evaluate different antibiotic prophylaxis strategies; therefore, a quantitative network comparison based on the Bayesian theorem was necessary. This theorem incorporates both direct and indirect information through a common comparator to obtain estimates of the relative interventional effects on multiple intervention comparisons.^[14,15]^ The values of surface under the cumulative ranking curve (SUCRA) probabilities based on the consistency model were presented to clarify the pros and cons of different strategies. The highest SUCRA values represented the probability of achieving the best clinical effects regarding respective parameters.^[16,17]^ Odds ratios (ORs) and related 95% credible intervals (CIs) derived from the network meta-analysis were calculated to compare different strategies, and publication bias was assessed by examining the funnel plot symmetry. Meanwhile, sensitivity analysis was also conducted to test the reliability of the main results. However, a pairwise meta-analysis was also conducted if additional evidence was needed. In this condition, heterogeneity (*I*^2^ index statistic) in the study design was used to estimate a data mode using fixed (*I*^2^ < 50%) or random (*I*^2^ > 50%) effects models.^[18]^ The associated 95% CIs were calculated, and the level of statistical significance was set at *P* < .05. Data manipulation, statistical analyses of network meta-analysis, and pairwise analyses were conducted using the Aggregate Data Drug Information System automated software (ADDIS, Version 1.16, GZ Groningen, Netherlands) and the Stata software package (Version 12.0, StataCorp LLC, Texas).

## 3 Results

### 3.1 Study characteristics and quality assessment

**Figure 1:**
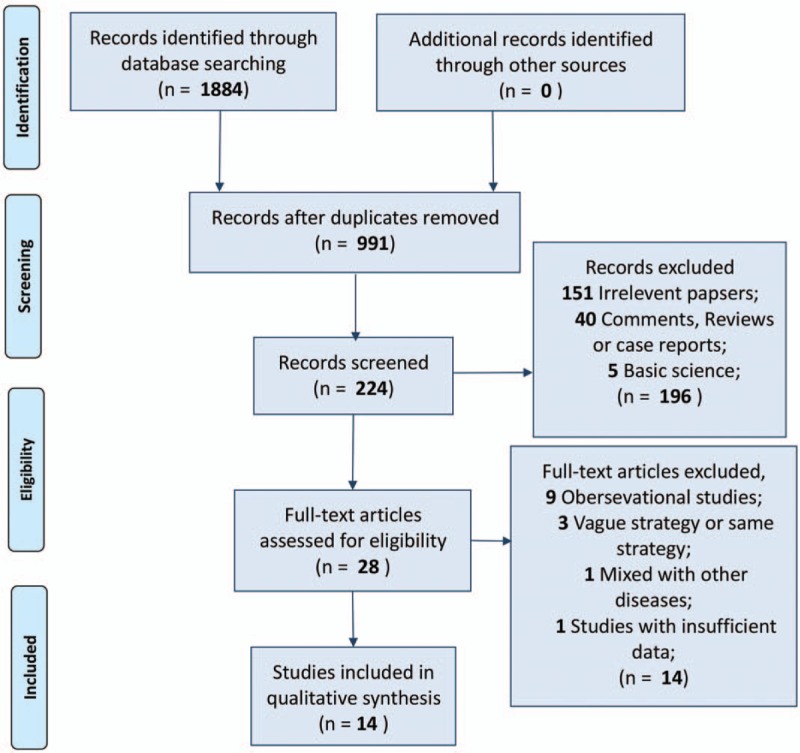
Flow diagram of the process of selecting studies for the current network meta-analysis.

**Figure 2:**
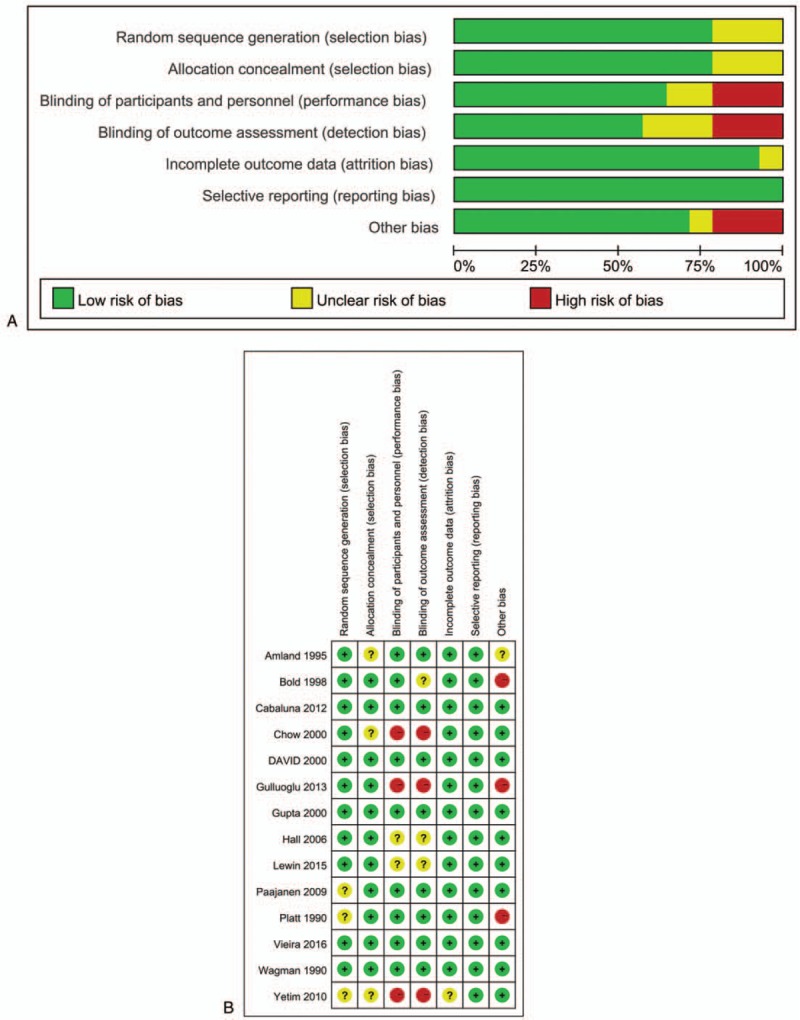
Bias assessment for included trials. (A) Risk of bias graph is presented as percentages across all of the included studies; (B) Judgments regarding each risk of bias item for each included study.

After initially identifying 1884 relative studies through a systematic retrieval, we included a total of 14 RCTs containing 3713 patients for quantitative comparison^[19–32]^ (Fig. 1). Among all included trials, 4 strategies, namely, preoperative application (PRA, defined as administration before skin incision), intraoperative application (INA, defined as the period from skin incision to closure), postoperative application (POA, defined as any period after skin closure), negative control (NC, defined as placebo administration or no antibiotic intervention), and their combinations were addressed (Table 1).^[19–22,24,26–32]^ For quality assessment, most of the included trials reported random consequence generation and allocation concealment. Meanwhile, more than half of the included trials were based on a double-blind procedure. Thus, in general, the overall quality was rated as high grade (Fig. 2).

**Table 1:**
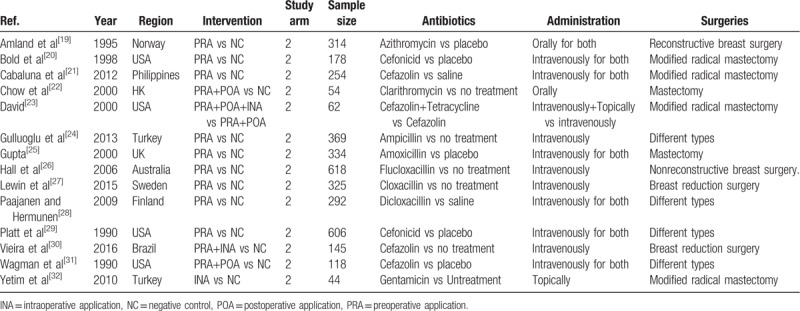
Characteristics of included studies.

### 3.2 Results of the network meta-analysis

**Figure 3:**
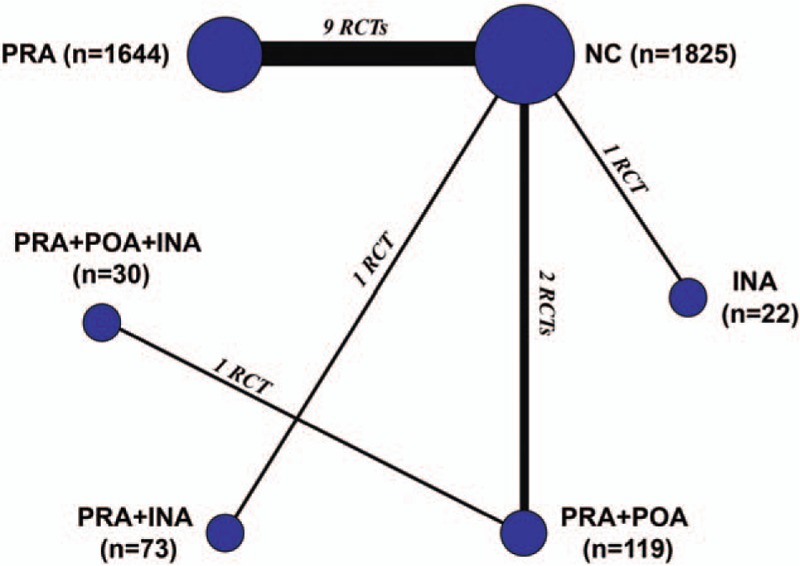
Network connections of all of the included trials. The numbers on the line indicate the quality of studies compared with every pair of strategies, which are also represented by the width of the lines. In addition, the sizes of the areas of the circles indicate the respective sample sizes. INA = intraoperative application, NC = negative control, POA = postoperative application, PRA = preoperative application.

**Figure 4:**
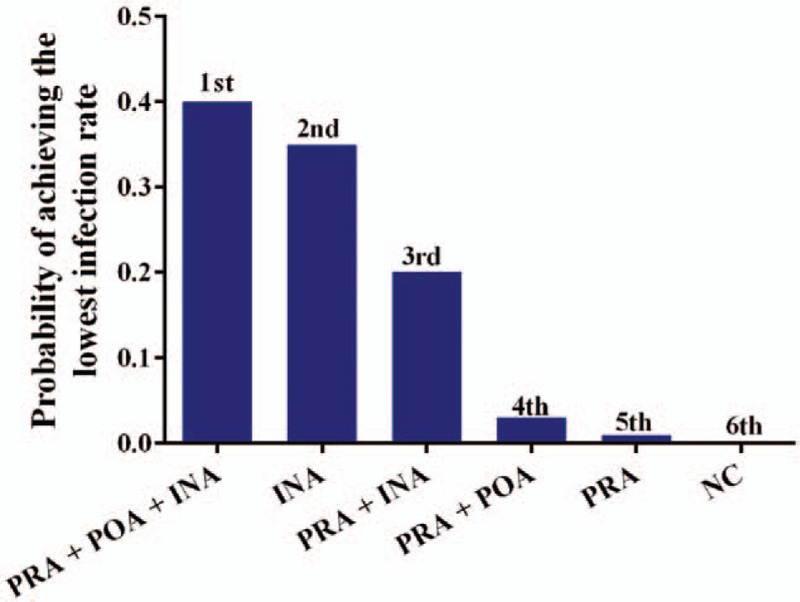
Histogram of probability of achieving the lowest infection rate regarding included strategies.

We performed quantitative pooled estimations based on the network connections of the included trials regarding the postoperative infection rate. All 14 included trials reported 6 different treatment strategies with different sample distributions (Fig. 3). After quantitative calculation based on the Bayesian theorem was done, the results indicated that PRA plus POA plus INA (SUCRA, 0.40), which was followed by INA alone (SUCRA, 0.35) and PRA plus INA (SUCRA, 0.20), revealed the highest probability of achieving the best clinical effects on reducing postoperative infections (Fig. 4) (Supplementary Table S2). Therefore, the combined application of PRA plus POA plus INA was the superior antibiotic prophylaxis strategy for breast surgeries.

### 3.3 Sensitivity analysis and publication bias

**Figure 5:**
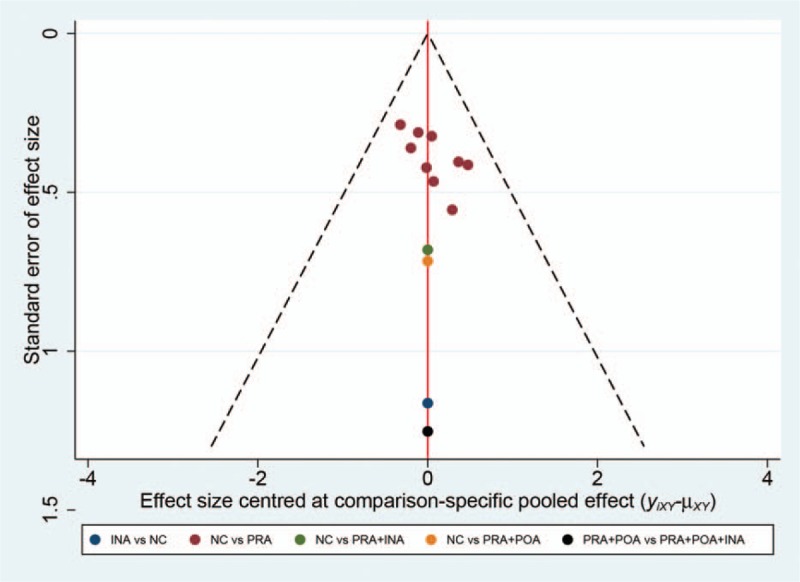
Publication bias regarding postoperative infections tested by a funnel plot.

To ensure the reliability of the main results, we conducted sensitivity analyses based on the following issues: omitting oral administration of antibiotics and omitting trials that were published more than 2 decades ago. After omitting the trials with oral administration of antibiotics (Supplementary Figure S1), 12 trials containing 3345 cases were pooled and estimated, and the results indicated that PRA plus POA plus INA still possessed the highest probability of being the best strategy (SUCRA, 0.52), followed by INA (SUCRA, 0.32) and PRA plus INA (SUCRA, 0.14) (Supplementary Figure S2) (Supplementary Table S3). Meanwhile, 10 trials containing 2497 patients were selected after reserving publications within the past 2 decades (Supplementary Figure S3). Similarly, we detected the same rank of included strategies, PRA plus POA plus INA (SUCRA, 0.46), INA alone (SUCRA, 0.24), and PRA plus INA (SUCRA, 0.15), compared with the main results (Supplementary Figure S4) (Supplementary Table S4). On the contrary, publication bias was detected by a funnel plot. The results indicated that no obvious publication bias existed in our study based on the funnel plot symmetry (Fig. 5). Therefore, we determined that the main results of the current study wre reliable.

### 3.4 Quality of evidence

On applying GRADE to findings from the network meta-analysis combining direct and indirect evidence, we rated the evidence from current network meta-analysis. There were 5 direct and 15 indirect comparisons and we noted that low or very low evidence were rated for most relative comparisons (Supplementary Table S5).

### 3.5 Additional analysis

We performed a network meta-analysis and identified superior antibiotic prophylaxis strategies for breast surgeries. However, the results of Bayesian calculations still needed to be validated at the statistical level. Therefore, we statistically evaluated the respective strategies by conducting pairwise pooled estimations. First, we performed a direct comparison between the antibiotic prophylaxis arm and the NC arm. On the basis of the fixed model (*I*^2^ = 37.4%), the result revealed that antibiotic prophylaxis significantly reduced the postoperative infection rate [OR (95% CI) = 0.57 (0.45–0.72); Test *Z* = 4.83; *P* = .000] (Supplementary Figure S5). Thus, we concluded that antibiotic prophylaxis was effective for reducing postoperative infections. Meanwhile, as mentioned above, pooled estimations of 6 different strategies indicated that PRA plus POA plus INA revealed the best clinical effects. To validate this result statistically, we conducted a pairwise meta-analysis by comparing PRA plus POA plus INA versus other strategies. However, only 1 RCT reported the PRA plus POA plus INA strategy; therefore, a direct meta-analysis could not be conducted. On the contrary, as previously described, both in the main results and the sensitivity analysis, the top 3 superior strategies were the same rank order, and interestingly, all of them were related to INA. Therefore, we deduced that the crucial fact reducing postoperative infection was the effects of INA-related strategies. To statistically verify our speculation, direct comparisons were conducted between the INA-related arm and the non-INA related arm, and the results showed that the INA-related strategies showed significant clinical benefits [OR (95% CI) = 0.28 (0.10–0.80); Test *Z* = 2.38; *P* = .017] (Supplementary Figure S6). In addition, the potential best strategy, PRA plus POA plus INA, was also related to PRA and POA; thus, we performed additional comparisons to statistically elucidate the roles of PRA and POA with similar procedures. After pooled estimations, it was demonstrated that PRA-related strategies significantly reduced the postoperative infection rate [OR (95% CI) = 0.61 (0.50–0.75); Test *Z* = 4.70; *P* = .000] (Supplementary Figure S7), while POA did not [OR (95% CI) = 0.40 (0.10–1.62); Test *Z* = 1.29; *P* = .198] (Supplementary Figure S8).

## 4 Discussion

Breast surgery, without prior infection, without penetration of the respiratory, gastrointestinal, or urinary tracts, and with primary suture closure, was thereby classified as a clean operation. Theoretically, the operation should not require antibiotic prophylaxis.^[33]^ However, many studies revealed high postoperative infection rates, which made antibiotic prophylaxis necessary for this clean operation, although the debate of using antibiotic prophylaxis still remains. Due to the miscellaneous antibiotic prophylaxis strategies that have been widely applied in the clinic, the current study estimated the superior strategy by a network meta-analysis based on the Bayesian theorem. We first demonstrated that PRA plus POA plus INA revealed the best clinical benefit by possessing the highest probability of the lowest postoperative infection rate by the pooled estimation of 14 RCTs containing 6 different strategies. However, this result could not be validated at the statistical level due to inadequate relative trials. On the contrary, we noticed that both regarding the main result and the sensitivity analysis, the top 3 superior strategies were all the same and all were INA-related. Thus, we deduced that the INA-related strategy seemed playing a crucial role in reducing postoperative infections, and the results of the pairwise meta-analysis demonstrated our speculation with significant differences. We knew that the intraoperative period was the only stage with surgical site exposure. Thus, this strategy was combined with many surgical factors influencing postoperative infection. Meanwhile, intraoperative administration was often based on topical application. This procedure may not increase renal and hepatic burden and impairment, lower the risk of developing resistant pathogens, or have the capacity to kill bacteria by direct interaction.^[34,35]^ These facts may reveal that intraoperative antibiotic prophylaxis effectively reduces postoperative infection. On the contrary, our results also determined that preoperative administration was effective at the statistical level with adequate evidence. Preoperative prophylactic antibiotics are recommended in many surgical disciplines to reduce infection-related morbidities and costs, but clean surgical procedures have not favored the routine use of antibiotics prophylactically.^[36–38]^ Meanwhile, unlike major organ resection, breast surgeries bring less systematic burdens, which make patients susceptible to infection. Therefore, antibiotic prophylaxis was not regularly recommended at the beginning. However, breast surgery involves the skin, fat, glands, and ducts, which could all be infected at different rates. Thus, systematic anti-infection prophylaxis is still needed. Preoperative administration of antibiotics is often performed before skin incision for approximately 2 hours, which may cause peak blood concentrations to occur during the intraoperative period. Thus, all these facts may be the potential reasons why preoperative and intraoperative had clinical effects.

To our knowledge, the current study is the first network meta-analysis to evaluate different antibiotic prophylaxis strategies based on the Bayesian theorem. A previous review claimed that there was no evidence to support any antibiotic prophylaxis strategies to reduce postoperative infections for breast surgery.^[5]^ Then, this conclusion was updated by 2 recent pairwise meta-analyses, which determined that antibiotic prophylaxis was effective by simply comparing experimental and control arms^[39,40]^ and prolonged application seemed to have no clinical benefit based on insufficient high-quality raw data.^[41]^ However, all 4 publications failed to perform a quantitative network estimation, and their classifications of strategies lacked accurateness and meticulousness, which may not reveal the essential roles of different strategies. And these publications basically only payed attention on PRA. More importantly, some recent trials were not included at that time, and the authors did not raise explicit future research directions. Therefore, it was necessary to perform a comprehensive network evaluation for different antibiotic prophylaxis strategies with accurate descriptions. And the current study implied that intraoperative antibiotics administration may also play a crucial role as PRA did. Unlike those simple pairwise meta-regression analyses, our study made a comprehensive assessment of all reported antibiotic prophylaxis strategies with accurate descriptions and classifications. We discovered that preoperative- and intraoperative-related antibiotic administration strategies were effective. However, some pure administration strategies still need to be evaluated; thus, relative high-quality trials are still needed.

### 4.1 Limitations

In the current study, we evaluated and determined the role of different antibiotic prophylaxis strategies by network and pairwise meta-analyses for the first time. Nevertheless, we must admit that some limitations exist in our study. First, although 14 RCTs were included, most of them had short pre- and intraoperative durations. Thus, the studies focused on POA (such as long-duration postoperative administration) were inadequate. Thus, our conclusions need to be further validated in duplicate. Second, although our results supported that the combination of PRA plus INA plus POS reveals superior benefit, pairwise comparison could not be conducted due to inadequate trails. Moreover, although we provided new evidences in this field, some important facts still need to be further addressed (the role of pure INA, for instance). Meanwhile, according to GRADE items, most of evidence from current meta-analysis were rated low or very low. We speculated that was associated with included samples, uncertain direct evidence, and wide confidence intervals. That was why more high-quality trails were needed regarding some strategies. Third, postoperative infections could also be influenced by surgical techniques, specific antibiotic doses, different surgery types, and wound managements. These confounding factors may have contributed to our results. Finally, we only focused on the infection rate as parametric data without comparing other parameters, such as antibiotic-related adverse events, due to insufficient raw data. This is another reason why more trials are urgently needed.

### 4.2 Future directions

Notably, although PRA- and INA-related strategies were demonstrated to be effective according to our results, however, the essential role of pure INA strategy was still unknown due to insufficient raw data and whether they should be combined with a POA could not be determined due to the lack of relative trials. In addition, for pre- and intraoperative administrations, whether their combinations revealed better benefits or resulted in adverse effects is still unknown. Finally, whether pure postoperative administration could reveal similar clinical effects compared with PRA still needs to be elucidated. Therefore, trails investigating on PRA versus INA or their combinations versus pure individuals was needed. Moreover, the role of pure INA, POA, or relevant combinations also needs to be validated. Thus, we raised all these undetermined issues for future investigations.

## 5 Conclusion

On the basis of the results of network meta-analysis and pairwise comparison, we determined that even with the development of asepsis and surgical procedures, antibiotic prophylaxis still played an important role in breast surgeries and application of antibiotics administered during pre-plus post-plus intraoperative periods seemed to reveal superior benefits. Moreover, according to current evidence, preoperative- and intraoperative-related strategies were statistically determined effective and pure PRA of antibiotic prophylaxis played an important role. However, the role of intraoperative and postoperative administrations still needs to be further validated. Therefore, more high-quality trials are still needed in the future.

## Author contributions

**Conceptualization:** Zhisu Liu, Zhen Li.

**Data curation:** Tao Guo, Baiyang Chen, Fengying Rao.

**Formal analysis:** Ping Wu.

**Investigation:** Tao Guo, Ping Wu.

**Methodology:** Fengying Rao, Ping Wu, Pengpeng Liu.

**Project administration:** Zhisu Liu, Zhen Li.

**Resources:** Baiyang Chen, Ping Wu, Pengpeng Liu.

**Software:** Tao Guo, Baiyang Chen, Fengying Rao, Pengpeng Liu.

**Validation:** Ping Wu, Pengpeng Liu.

**Visualization:** Ping Wu.

**Writing - original draft:** Tao Guo, Zhen Li.

**Writing - review & editing:** Tao Guo, Zhen Li.

## Supplementary Material

Supplemental Digital Content
