# Supplementary material for: Identifying the superior antibiotic prophylaxis strategy for breast surgery: A network meta-analysis
Source: Medicine (Baltimore). 2019 Apr 26;98(17):e15405. doi: 10.1097/MD.0000000000015405 (PMC6831324; doi:10.1097/MD.0000000000015405)

Supplementary Tables and Figures

Table S1. Example search strategy and process in MEDLINE.

# No.	Search	Results
1	exp (breast surgery) OR breast operation/	126233
2	exp mastectomy/	36937
3	1 OR 2	130536
4	exp (prospective) OR random/	1181338
5	exp randomized controlled trial/	609584
6	4 OR 5	1646110
7	exp (prophylactic antibiotic) OR antibiotic prophylaxis/	27199
8	3 AND 6	15124
9	7 AND 8	75

Table S2. Comparisons of included strategies in network meta-analysis regarding infection. Odds ratios are presented in the cells in common between the column-defining and row-defining strategies. [OR: odds ratio; CI: credible intervals].

Comparison [OR (95%CI)]					
INA	6.14 (0.57, 180.71)	3.52 (0.32, 106.31)	1.51 (0.07, 59.31)	2.27 (0.11, 85.96)	0.93 (0.01, 97.45)
0.16 (0.01, 1.76)	NC	0.57 (0.37, 0.87)	0.24 (0.03, 1.24)	0.38 (0.06, 2.02)	0.15 (0.00, 4.03)
0.28 (0.01, 3.13)	1.75 (1.15, 2.73)	PRA	0.42 (0.06, 2.40)	0.66 (0.10, 3.89)	0.26 (0.00, 7.38)
0.66 (0.02, 15.27)	4.16 (0.81, 29.46)	2.37 (0.42, 17.70)	PRA+INA	1.51 (0.13, 20.41)	0.62 (0.01, 27.87)
0.44 (0.01, 8.99)	2.66 (0.49, 17.66)	1.53 (0.26, 10.40)	0.66 (0.05, 7.98)	PRA+POA	0.41 (0.01, 6.69)
1.08 (0.01, 107.64)	6.70 (0.25, 358.61)	3.82 (0.14, 200.87)	1.60 (0.04, 105.93)	2.43 (0.15, 78.44)	PRA+POA+INA

Table S3. Sensitivity analysis by omitting orally applications. [OR: odds ratio; CI: credible intervals].

Comparison [OR (95%CI)]					
INA	6.64 (0.62, 132.66)	4.42 (0.40, 85.72)	1.49 (0.08, 41.25)	2.23 (0.12, 67.75)	0.58 (0.00, 54.45)
0.15 (0.01, 1.61)	NC	0.66 (0.45, 0.93)	0.23 (0.04, 0.99)	0.35 (0.07, 1.73)	0.09 (0.00, 1.65)
0.23 (0.01, 2.51)	1.51 (1.07, 2.20)	PRA	0.35 (0.06, 1.59)	0.52 (0.10, 2.72)	0.14 (0.00, 2.55)
0.67 (0.02, 12.65)	4.34 (1.01, 25.24)	2.88 (0.63, 16.74)	PRA+INA	1.61 (0.17, 19.36)	0.39 (0.01, 16.12)
0.45 (0.01, 8.31)	2.89 (0.58, 14.64)	1.91 (0.37, 9.97)	0.62 (0.05, 5.91)	PRA+POA	0.25 (0.01, 2.87)
1.71 (0.02, 235.44)	11.18 (0.61, 623.85)	7.38 (0.39, 397.80)	2.59 (0.06, 188.53)	3.93 (0.35, 163.49)	PRA+POA+INA

Table S4. Sensitivity analysis by reserving trails published within 2 decades. [OR: odds ratio; CI: credible intervals].

Comparison [OR (95%CI)]					
INA	5.90 (0.58, 211.66)	4.04 (0.39, 147.83)	1.54 (0.07, 68.31)	0.08 (0.00, 50540447530.93)	0.02 (0.00, 23501446336.02)
0.17 (0.00, 1.72)	NC	0.70 (0.42, 1.09)	0.25 (0.04, 1.23)	0.01 (0.00, 6091569839.88)	0.00 (0.00, 3261865144.05)
0.25 (0.01, 2.55)	1.43 (0.91, 2.38)	PRA	0.35 (0.05, 1.93)	0.02 (0.00, 7705975107.61)	0.01 (0.00, 4447088899.81)
0.65 (0.01, 14.80)	4.07 (0.81, 26.30)	2.84 (0.52, 18.33)	PRA+INA	0.05 (0.00, 36435450125.84)	0.02 (0.00, 17002668732.31)
12.81 (0.00, 6270941489852186.00)	73.19 (0.00, 32134055490432668.00)	49.52 (0.00, 22847173754293744.00)	21.09 (0.00, 9748916957991122.00)	PRA+POA	0.42 (0.01, 7.53)
44.52 (0.00, 11906801619902376.00)	217.74 (0.00, 88723697869694848.00)	148.00 (0.00, 64194058868728304.00)	62.61 (0.00, 26613010474277408.00)	2.41 (0.13, 83.64)	PRA+POA+INA

Table S5. Overall GRADE Quality of Evidence From Network Meta-analysis

Applied Strategy	Quality of Evidence
**Compared with NC**	
INA	Very low
PRA+POA	Moderate
PRA+INA	Low
PRA+POA+INA	Very low
PRA	High
**Compared with INA**	
PRA+POA	Very low
PRA+INA	Very low
PRA+POA+INA	Very low
PRA	Low
**Compared with PRA+POA**	
PRA+INA	Low
PRA+POA+INA	Very low
PRA	Low
**Compared with PRA+INA**	
PRA+POA+INA	Very low
PRA	Low
**Compared with PRA+POA+INA**	
PRA	Low

Figure S1. Network connections of included studies with the available direct comparisons by omitting orally applications. The size of circles and width of lines respectively represent the number of included sample sizes and trails. PRA: preoperative application; POA: postoperative application; INA: intraoperative application; NC: Negative control.

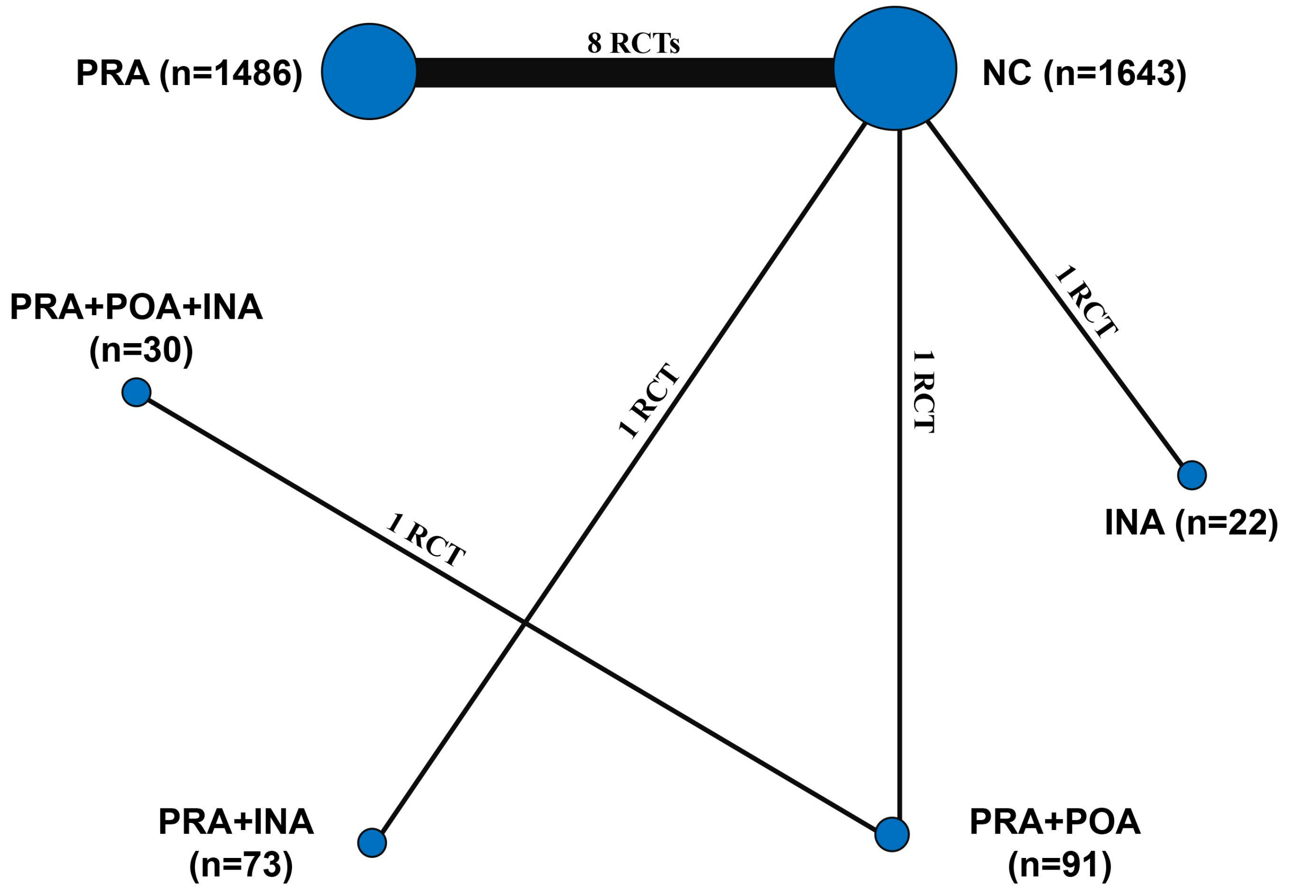

Figure S2. Probability of achieving the lowest infection rate regarding included strategies by omitting orally applications. PRA: preoperative application; POA: postoperative application; INA: intraoperative application; NC: Negative control.

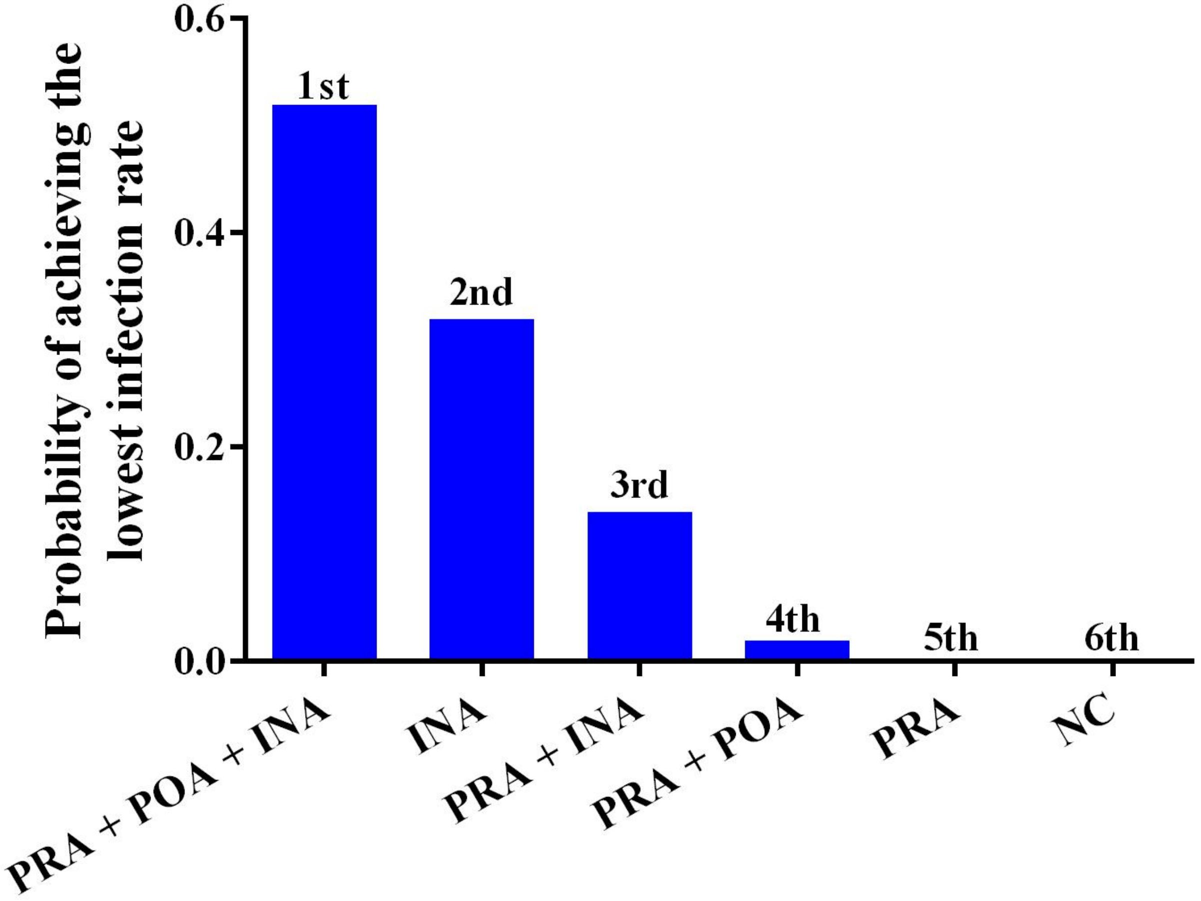

Figure S3. Network connections of included studies with the available direct comparisons by reserving trails published within 2 decades. The size of circles and width of lines respectively represent the number of included sample sizes and trails. PRA: preoperative application; POA: postoperative application; INA: intraoperative application; NC: Negative control.

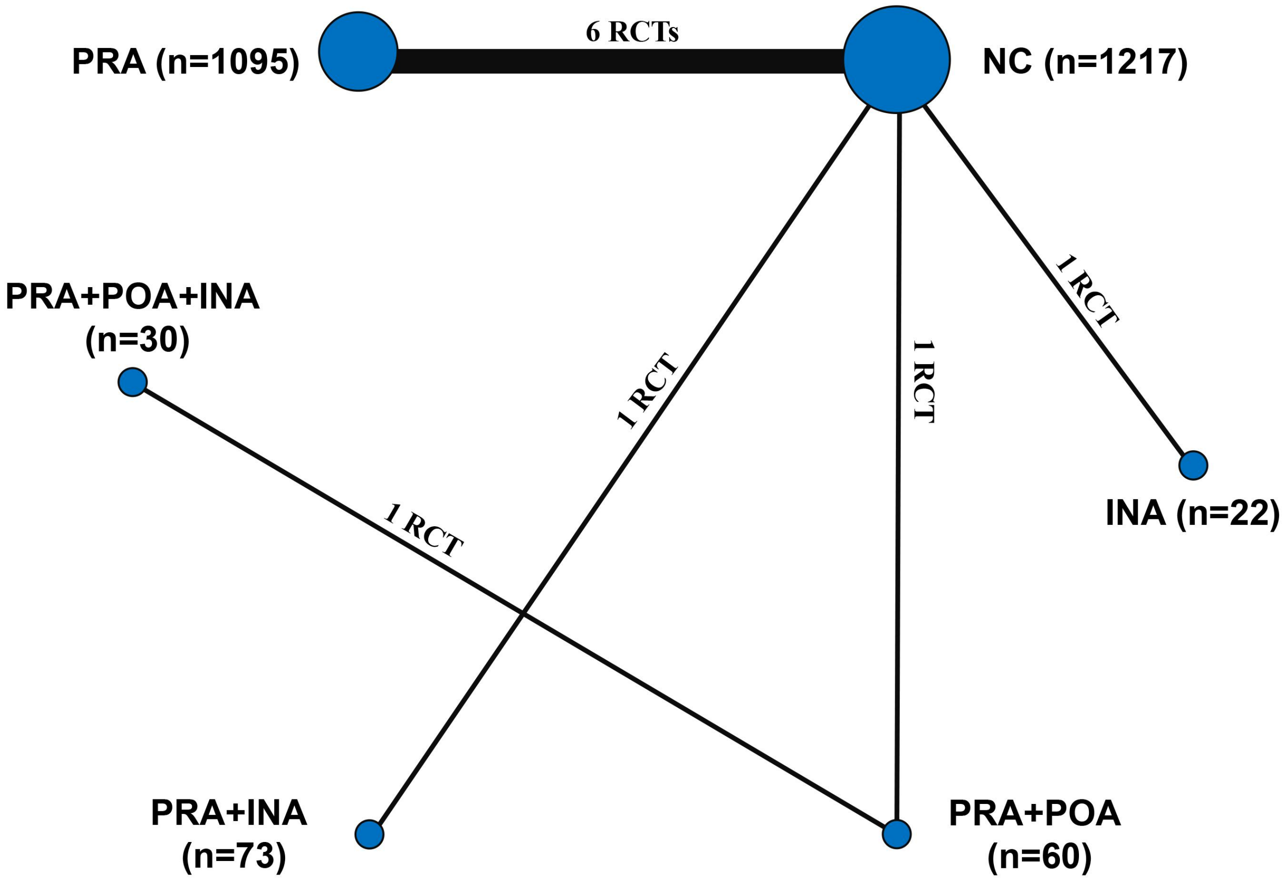

Figure S4. Probability of achieving the lowest infection rate regarding included strategies by reserving trails published within 2 decades. PRA: preoperative application; POA: postoperative application; INA: intraoperative application; NC: Negative control.

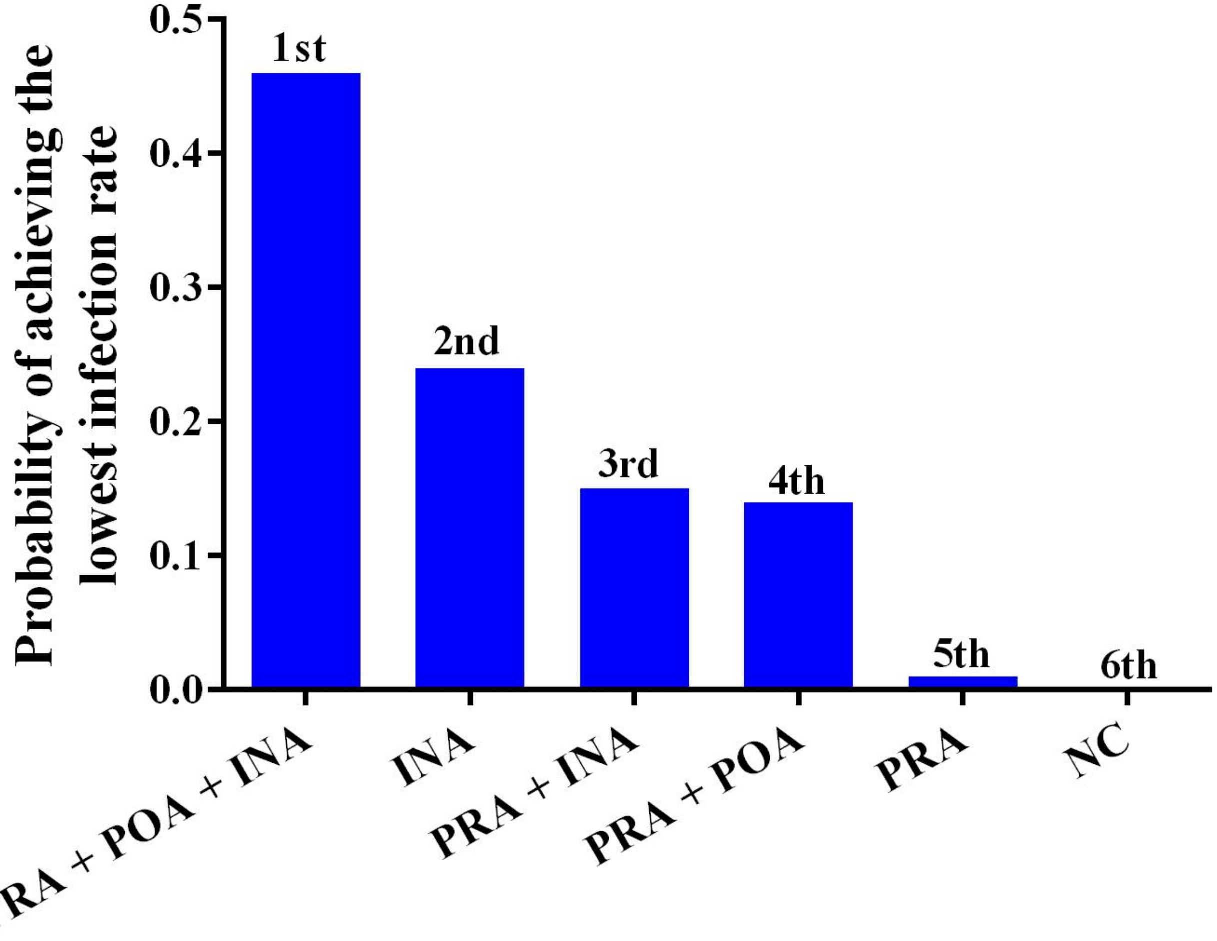

Figure S5. Pair-wised meta-analysis between antibiotic prophylaxis group and control group.

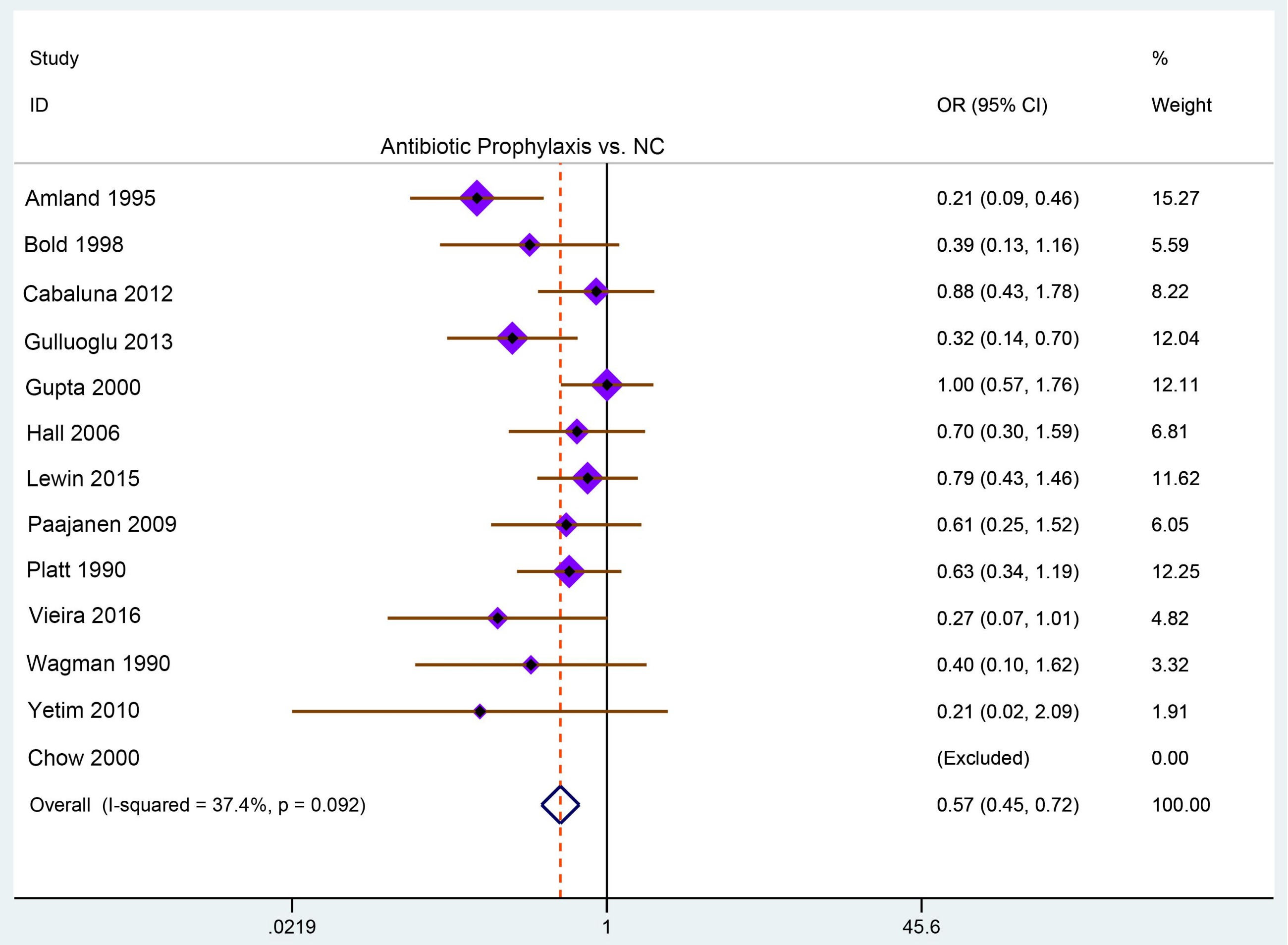

Figure S6. Pair-wised meta-analysis between intraoperative antibiotic application group and non-intraoperative antibiotic application group.

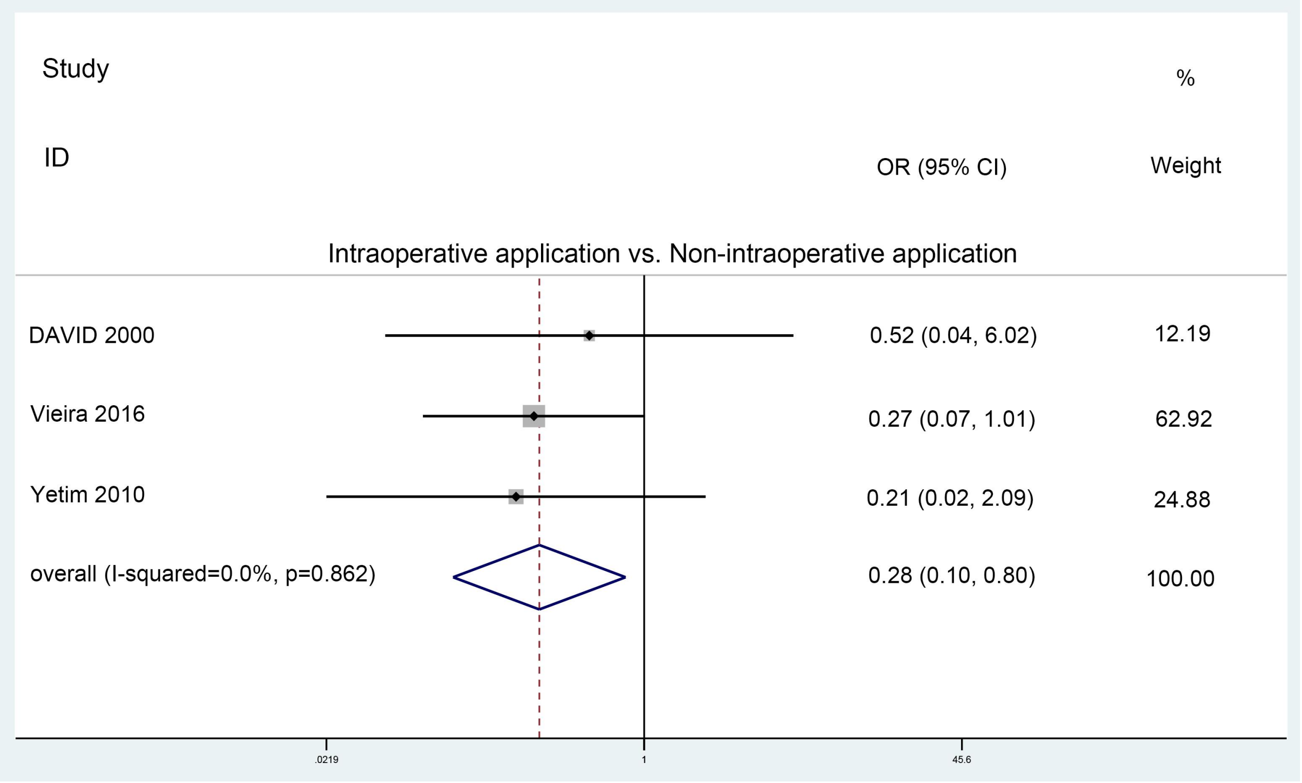

Figure S7. Pair-wised meta-analysis between preoperative antibiotic application group and non-PRA group.

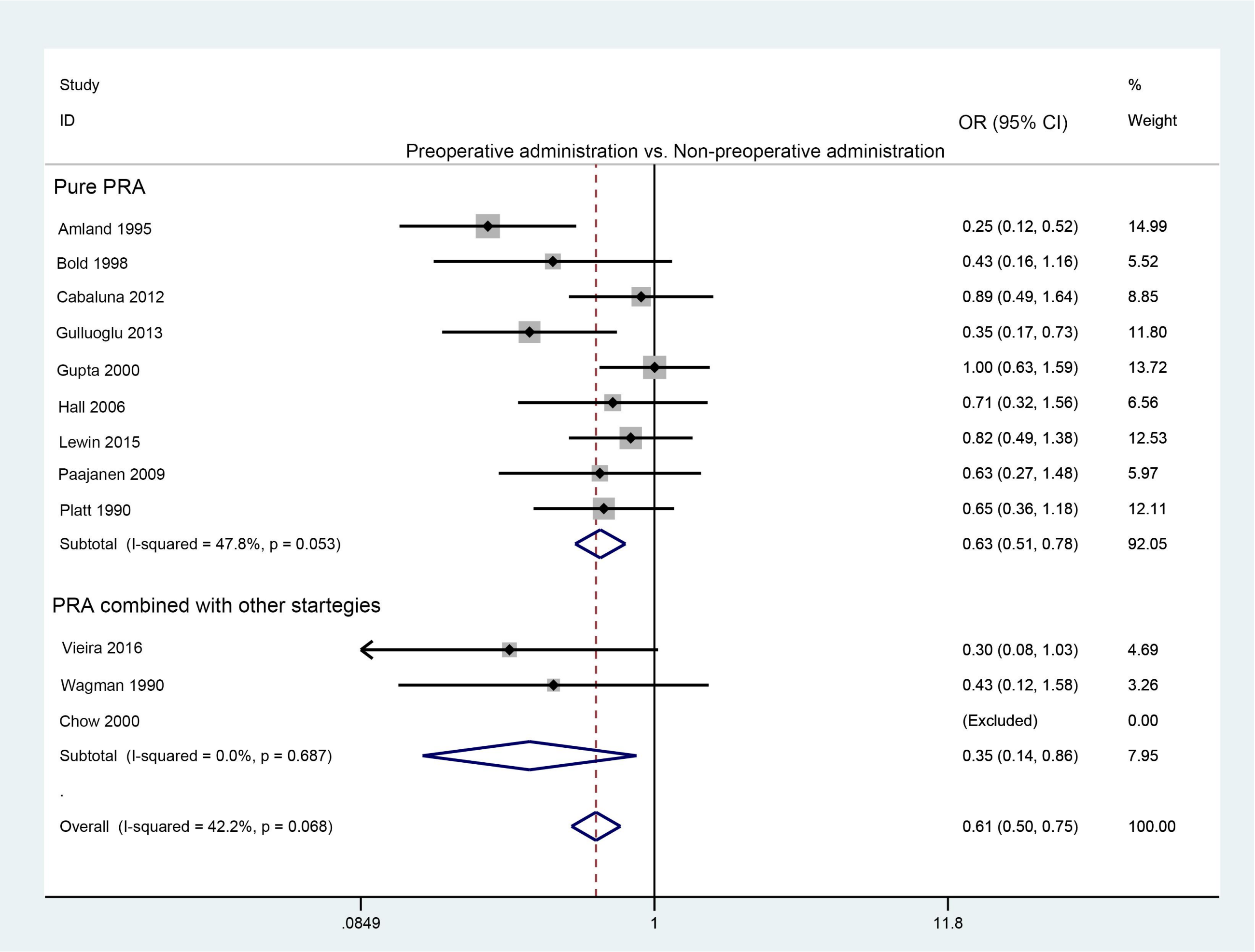

Figure S8. Pair-wised meta-analysis between postoperative antibiotic application group and non-postoperative antibiotic application group.

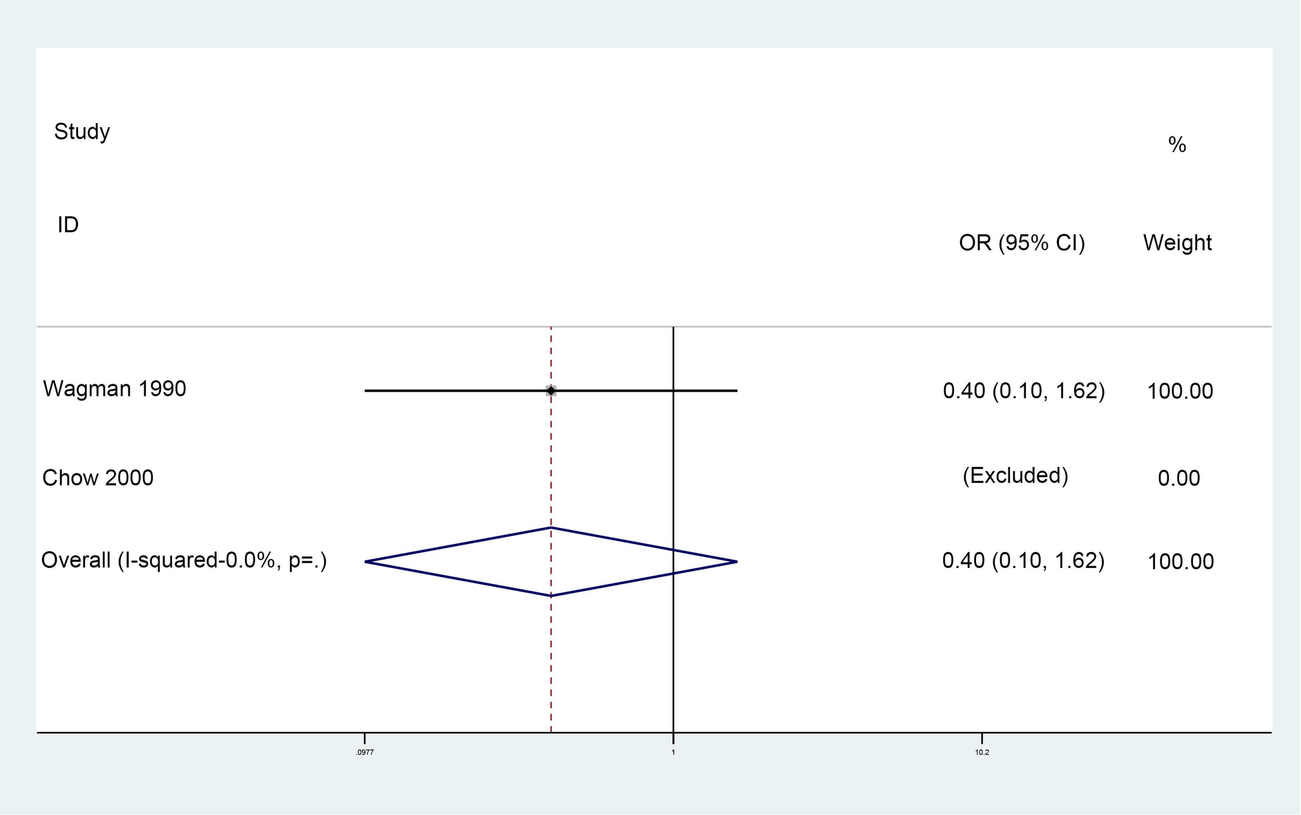

Supplement: Supplemental Digital Content [file medi-98-e15405-s001.pdf]
